# Supplementary material for: Can intersectionality help with understanding and tackling health inequalities? Perspectives of professional stakeholders
Source: Health Res Policy Syst. 2021 Jun 25;19:97. doi: 10.1186/s12961-021-00742-w (PMC8227357; doi:10.1186/s12961-021-00742-w)
Supplement: Supplementary file 3 — Additional file 3. Workshop summary. [file 12961_2021_742_MOESM3_ESM.pdf]

Report of workshop: Can intersectionality help with understanding and tackling health inequalities? Perspectives from research, policy and practice

Workshop for the ESRC funded project 'Chronic disease and healthy ageing at the intersections'
INOX, Level 5, Students' Union Building, Sheffield S10 2TG
May 20th 2019

Contents – add executive summary?

1. Introduction, background and aims
2. Summary of participants' notes
3. Whole group feedback from subgroup discussions
4. Whole group feedback on next steps
5. Participant list and email addresses
6. Presentations

1. Introduction, background and aims

A workshop was held at the University of Sheffield on May 20th 2019 on the topic of intersectionality in health inequalities research, organised by Dr. Daniel Holman, Professor Sarah Salway, and Dr. Andy Bell as part of the ESRC funded project 'Chronic disease and healthy ageing at the intersections'. The audience were a mixture of academics, patient representatives, third sector organisations, local city councils and other national and local bodies.

The purpose of the workshop was to facilitate an exchange around the idea of intersectionality, which although 'jargony' sounding, is at heart the simple idea that social attributes such as gender, age, ethnicity, socioeconomic position, disability, sexuality, etc., *together* shape inequalities. Intersectionality is currently becoming very popular in health inequalities research, so now is the ideal time to get input from people who have expertise in this area to 'sense check' how it is being and should be implemented. To do this, we purposively invited people from a range of backgrounds, the idea being that (i) academics should be guided by the concerns of those from policy/practice, and (ii) these stakeholders might also consider the relevance of intersectionality to their own work.

The specific aims of the workshop were as follows:

- To spread awareness of intersectionality and potential for understanding/tackling health inequalities.
- To share examples of how intersectionality can be applied to health inequalities research.
- To get input from both academic and non-academic audiences on current challenges/issues in intersectionality research.
- To establish ways forward and next steps in promoting intersectional ways of thinking.
- To facilitate networking on this topic.

The morning session focussed on understanding and explaining inequalities, and the afternoon session focussed on policy responses, with the format of both sessions comprising three presentations followed by subgroup discussions. Finally, there was a wrap-up session where the subgroups fed back to the whole group, and a brief whole group reflection on ideas and issues raised during the day, as well as potential next steps.

2. Summary of participants' notes

For each of the presentations, participants were asked write on 'post-it' notes (i) one idea, concept, method, technique etc. that they thought might be useful in their own work (ii) one challenge or barrier with implementing this. Participants were also asked to note down ideas in the subgroup discussions. These notes are now summarised.

Broadly, participant notes fell under three areas – (i) knowledge and understanding (ii) methods and data, and (iii) policy and implementation.

Knowledge and understanding: Numerous participants made points about what intersectionality actually is, and how it should be conceptualised. It was noted that intersectionality is not a hypothesis, but a framework. It is a thinking tool rather than a solution. It is not only concerned with differential outcomes e.g. by population subgroup, but also inputs i.e. access to resources. Applying the concept to health inequalities exposes the need to think carefully about what we mean by health inequalities e.g. whether we are talking about equality or equity. One participant noted that structure and power are at the root of inequality, not the social groups through which these operate. Another suggested that public health and intersectionality are concerned with social justice, suggesting an underlying compatibility. A crucial dimension here is the need to incorporate an understanding of social power into intersectionality approaches, for example by considering how discrimination and social institutions influence health.

Methods and data: There is a sample size issue when considering granular subgroups. One potential answer is to use administrative data e.g. health records (though this brings its own challenges). Relatedly, datasets are very limited for certain attributes e.g. sexuality or ethnicity, especially when considered together with others such as age. Numerous participants made comments on mixed methods, and suggested they might help understand the meaning of intersectional differences. This can also help mitigate the risk of stigmatising intersectional groups. A distinction made in intersectionality is between intercategorical approaches – comparing various subgroups, and intracategorical – focussing within particular subgroups e.g. the experiences of retired ethnic minority men. A challenge here is that people do not generally think of their bodies/health problem as being related to their ethnicity, age, gender etc., but rather as a whole.

Other methodological issues raised included categorisation – who does it and with what implications. The difficulty of categorising age was noted in relation to the difference between biological and chronological age. Methods for measuring intersectionality and explaining outcomes need to consider events, contexts and processes. As well as adversity, protective factors are also important e.g. related to multigenerational families. There is a need to move away from description towards explanation, and unpack causal processes. This is useful in order to focus on factors amenable to intervention (see policy section below), and follows the point that evidence from one group does not necessarily apply to another.

Policy and implementation: There is a need to identify modifiable factors, and potential implications for action should be kept in mind. Intersectional approaches need to have relevance for health outcomes and improving these. One of the fundamental tensions here is between population level universal approaches and addressing subgroup needs. It was suggested that *not* targeting minority groups is akin to targeting majority groups. Localised and community-based approaches could be useful in terms of targeting, though this raises the question of other characteristics cutting across localities, making it a difficult sell for policy makers. Specific ideas were raised here about social prescribing which is compatible with an intersectionality approach

but there is a question on how we can support primary care to commission differently for this. Another idea suggested is to apply intersectionality to see who is not seeking healthcare to identity gaps.

Some barriers and challenges to policy makers engaging with intersectionality were raised. Policy makers like simple, universal interventions. They will need convincing of what intersectionality adds to approaches focussing on one attribute, such as gender or socioeconomic status, at a time. The language of intersectionality might also be a barrier as it sounds like academic jargon. Policy makers also require value for money, and so targeted approaches need to demonstrate this, and ideally be flexible and scalable. The point that policy already struggles to deal with multimorbidity suggests it might also struggle with intersectionality, given the similarity in considering multiple factors at once. Finally, the point that intersectionality may be difficult to implement under austerity was raised. The wider socio-political is therefore important.

3. <u>Whole group feedback from subgroup discussions</u>

- There is a need to target upstream at the level of structure.
- There is a gap between intersectionality telling us how different subgroups might exist and strategies that are impactful from a health perspective.
- Lived experienced is based on *both* an individual and a sub/group view – not just latter.
- There is a challenge in identifying groups and thinking of mechanisms. There is a need to balance mechanisms and lived experience.
- Geography likely plays a part, and there is a question of how much compared with socioeconomic factors.
- There is a need to not leave people out and intersectionality can help identify these.
- There are different levels of intersectionality and there is a question of how to integrate them.
- There is a need to keep a focus on power.
- We need to differentiate equality of access and equity of outcomes.
- There is the challenges of measuring impact and lack of measures in intersectionality.
- Discrimination is difficult to identify.

4. <u>Whole group feedback on next steps</u>

- Potential next steps include a mailing list, final project conference, a special issue, and co-authorship, involving both academics and non-academics. Participants to be asked.
- The Equality Act needs updating. It only allows consideration of one characteristic at a time.
- Shall we look at who is interested in this in politics? We could learn from them.
- We can draw from consideration of processes across the life course – there is lots of work already on this that is relevant to intersectionality.
- Just looking at subgroups is dangerous – we also need to move towards the social and political determinants of health.
- A Delphi type approach may be useful in deciding on ways forward.
- A focus on causes is needed in order to intervene.
- 5-10 points for how to embrace intersectionality would be useful.
- Using the term intersectionality may silo work in this area. May be better to frame it as health inequality research, best to talk of it as health inequality work with a specific method/framework.
- In the US intersectionality is built in as the next step, an advantage, a plus. In the UK existing well-known frameworks could be expanded in the way same.
- Intersectionality as layer upon layer of discrimination is easily understood.

- The obstacle isn't the name, it's putting an emphasis on structural inequalities and social justice over an individual approach.

5. <u>Participant list and email addresses</u>

[Retracted for website]

6. <u>Presentations</u>

Please get in touch if you would like to access the presentations below.

Dan Holman, Research Fellow, University of Sheffield
Welcome and Introduction

Greta Bauer, Professor of Epidemiology and Biostatistics, Western University Canada
Intersectionality in Health Inequalities Research

Mai Stafford, Principal Data Analyst, The Health Foundation
The Health Care Utilisation of People with Multiple Conditions

Brian Beach, Senior Research Fellow, International Longevity Centre UK
Health Inequalities Among Older LGBT People in the UK: New Insights and Implications

Jenny Douglas, Senior Lecturer in Health Promotion, The Open University
It's All About the Mix: Intersectionality- Informed Mixed Methods Research on Cigarette Smoking Among African Caribbean Young Women. Lessons for Policy and Practice in Public Health

Nuzhat Ali, National Lead Musculoskeletal Health and Wellbeing, Public Health England
Productive Healthy Ageing for all Requires a Targeted Public Health Approach

Jabeer Butt, Chief Executive, Race Equality Foundation
You Make it Sound Like a Death Sentence! Exploring Black and Minority Ethnic People's Experience of Dementia and Dementia Services
